# The treatment outcomes of tuberculosis among health care workers in a general hospital in the Mpumalanga province, South Africa

**DOI:** 10.4102/phcfm.v1i1.86

**Published:** 2009-09-15

**Authors:** Idongesit S. Ukpe, Julia Blitz, Jannie Hugo, Ralph Nkosi, Thembi Mpangane, Sharon McLaren

**Affiliations:** 1 Department of Family Medicine, University of Pretoria, South Africa; 2 Themba Hospital, Mpumalanga Province, South Africa; 3 National Health Laboratory Service, Mpumalanga Province, South Africa

**Keywords:** tuberculosis, treatment outcomes, health care workers, Themba Hospital, Mpumalanga province

## Abstract

**Background:**

South Africa is one of the countries in the world with a high burden of tuberculosis (TB). High rates of unfavourable treatment outcomes have remained a feature of TB control in the country. The objective of the current study was to examine the treatment outcomes of TB among health care workers (HCWs) at a rural general hospital in the Mpumalanga province of the country, as well as the clinical care that was provided to the HCWs. The purpose of the study was to identify possible areas for improvement in the TB care services provided to HCWs in the hospital, especially with regard to their clinical management.

**Method:**

The research described in this article consists of a retrospective descriptive study. Relevant data on HCWs diagnosed with TB in the hospital during 2007, the TB care services offered to the HCWs, and the treatment outcomes of the HCWs were captured from the occupational health and TB control programme registers at the hospital onto a data capture sheet for the study and were subsequently analysed manually.

**Results:**

Nine HCWs, eight females and one male, were diagnosed and treated for TB in the hospital during 2007, an incidence rate of 941/100 000. Their ages ranged from 39 to 54 years, with a mean age of 48 years. By occupation, the nine HCWs consisted of six nurses (67%), one porter (11%), one general assistant (11%), and one clerk (11%). Of those treated for TB, seven (78%) had smear- positive pulmonary TB (PTB) and two (22%) had extra-pulmonary TB (EPTB). TB culture and drug susceptibility testing (DST) was undertaken for only one HCW. The HIV status was known for only two (22%) of the nine HCWs under review. Neither of the two HCWs with EPTB had the diagnosis confirmed by bacteriological or histopathological method. The seven HCWs with smear- positive PTB achieved a cure, and the two HCWs with EPTB successfully completed treatment, resulting in a treatment success rate of 100% for the nine HCWs.

**Conclusion:**

The HCWs at Themba Hospital in the Mpumalanga province of South Africa who were diagnosed and treated for TB during 2007 all achieved favourable treatment outcomes. However, in view of the high rate of HIV/TB co-infection and the increasing problem of drug-resistant TB in the country, the clinical care provided to HCWs with TB by the hospital should be improved with regard to routine HIV counselling and testing and the routine early provision of DST. A protocol for the comprehensive management of HCWs with TB is currently undergoing development.

## INTRODUCTION

The risk of transmission of tuberculosis (TB), including drug-resistant TB, to health care workers (HCWs) in health care facilities where patients with the disease are diagnosed and treated is a recognised occupational hazard. Infection prevention and control procedures reduce, but do not completely eliminate, the risk of TB transmission in health care facilities.^1, 2, 3^ Studies in many countries with a high burden of TB have documented increased risk of TB infection and disease in HCWs, compared with the general population.^1^ The ongoing pandemic of human immunodeficiency virus (HIV) infection has further worsened the risk situation for HCWs.^2^

South Africa is burdened with a high rate of TB.^4, 5^ High rates of unfavourable treatment outcomes, such as defaulting from treatment, treatment failure and death, have remained a feature of the TB control programme in the country. In particular, defaulting from treatment was reported as the main barrier to reaching the internationally recommended treatment success target of 85% in the country.^4, 5^

In South Africa, unfavourable treatment outcomes have been shown to be significantly associated with increased risk for all forms of drug-resistant TB.^6^ A high prevalence of patients with unfavourable treatment outcomes could increase the risk of transmission of drug-resistant TB to HCWs in health care facilities. Between January 2004 and April 2007, 11 000 laboratory-confirmed multidrug-resistant TB (MDR-TB) cases were reported in the country.^4^ At least 6 000 newly active cases of MDR-TB have been reported in the country each year.^3^ Community and nosocomial transmission of MDR-TB is reported to have been confirmed in the country by epidemiological and molecular genetics studies.^6^

Drug-resistant TB, especially MDR-TB, is a recognised cause of failure of treatment with first-line anti-TB drugs.^3, 7 , 8^ Extensively drug-resistant TB (XDR-TB) carries very high mortality potential, especially among people with HIV/AIDS.^9^ The increased transmission of such forms of drug-resistant TB from patients to attending HCWs could result in an increased risk of unfavourable treatment outcomes, with cases of treatment failure and death among the HCWs concerned.

The unfavourable TB treatment outcome of defaulting from treatment is a major public health concern. The management of TB, therefore, lays much emphasis on measures to ensure adherence to treatment.^7^ Due to HCWs being at increased risk of contracting drug-resistant TB which, in turn, increases their risk of unfavourable treatment outcomes, additional special diagnostic and therapeutic measures for aggressive management of their TB have been recommended, in addition to measures aimed at ensuring very tight adherence to treatment.^3, 4 , 8^

This study examined the treatment outcomes of TB among HCWs in a general hospital in the Mpumalanga province of South Africa, as well as the clinical care that was provided to the HCWs. The purpose was to identify possible areas for improvement, especially in terms of clinical management, in the TB care services provided for HCWs in the hospital.

Themba Hospital is a regional general hospital situated in the Mbombela North subdistrict of the Ehlanzeni district in the province. The hospital has a bed capacity of 623 and a total staff complement of about 956. It provides the entire spectrum of general medical services, including special clinics for the treatment of those with HIV/AIDS and TB. About 68 cases of pulmonary tuberculosis (PTB) are diagnosed by sputum smear microscopy for acid-fast bacilli per month at the laboratory of the National Health Laboratory Service situated in the hospital.

The subdistrict is mainly rural and has a population of about 245 020 people. In 2007 the prevalence of TB in the population was estimated at 350/100 000, of which there were about 30 registered poly- and multidrug-resistant cases.

## METHOD

A research protocol was developed for a retrospective descriptive study to be undertaken into the treatment outcomes for HCWs who were diagnosed with, and treated for TB at Themba Hospital during 2007.

A data capture sheet was designed for the capturing of the relevant data by the TB control programme data capturer at the hospital from the occupational health and TB control programme registers kept at the hospital on HCWs who were diagnosed with TB during 2007. Details regarding the TB care services made available to them, and the treatment outcomes recorded for them on the TB control programme register were also captured.

Data capturing was conducted between November and December 2008, after obtaining ethical approval for the study from the Mpumalanga Provincial Research Ethics Committee, and the University of Pretoria Research Ethics Committee. The data were subsequently processed manually in February 2009.

The standardised TB control programme treatment outcome definitions of the categories of ‘cure’, ‘treatment completed’, ‘treatment failure’, ‘died’, ‘treatment interrupted or default’, and ‘transfer out’ were used (see Table 1), with the outcomes falling into the categories of ‘cure’ and ‘treatment completed’ being regarded as favourable, and those outcomes falling into the other categories being regarded as unfavourable.^7^

**TABLE 1:** TB treatment outcomes

TREATMENT OUTCOME	DEFINITION
Cure	Patient who is found to be sputum smear-negative in the last month of treatment, as well as on at least one previous occasion.
Treatment completed	Patient who has completed treatment, but who does not meet the criteria to allow for classification as a cure or failure.
Treatment failure	Patient who is sputum smear-positive at five months, or later during treatment. Also a patient who was initially smear-negative before starting treatment and who became smear-positive after completing the initial phase of treatment.
Died	Patient who dies, for any reason, during the course of treatment.
Treatment interrupted or default	Patient whose treatment was interrupted for two consecutive months or more.
Transfer out	Patient who has been transferred to another recording and reporting unit, and for whom the treatment outcome is not known.

## RESULTS

### Demography

A total of nine HCWs in the hospital were diagnosed with TB and put on treatment during the year 2007, an incidence rate of 941/100 000. The nine HCWs comprised eight females and one male. Their ages ranged from 39 years to 54 years, with a mean age of 48 years. By occupation, the nine HCWs consisted of six nurses (67%), one porter (11%), one general assistant (11%), and one clerk (11%).

### Site and the method of diagnosis of TB

Seven (78%) of the nine HCWs were diagnosed with PTB, and two (22%) with extra-pulmonary tuberculosis (EPTB). The seven HCWs with PTB were diagnosed as positive by means of sputum smear microscopy for acid-fast bacilli. The two HCWs with EPTB had tuberculous pleural effusion diagnosed by means of chest x-ray, microscopy and biochemical analysis of aspirated pleural fluid.

The TB culture and drug susceptibility test (DST) was undertaken for only one (11%) of the nine HCWs. The form of TB was indicated to be PTB, with the result of the test being recorded as both positive and sensitive.

### Patient category and treatment regimen

All nine HCWs were new patients according to the TB control programme patient category definition for patients who have not previously undergone treatment for TB, or who have taken anti-TB drugs for less than a month.^7^ All nine HCWs were treated with the recommended standardised fixed-dose combination first-line anti-TB drug regimen for new cases of TB in South Africa.^10^

### HIV status

HIV status was recorded for two (22%) of the nine HCWs as known, with the rest being unknown.

### Treatment outcomes

All nine HCWs successfully completed treatment. The seven HCWs who were diagnosed with PTB achieved a 100% favourable outcome, with a classification of ‘cure’. The two HCWs who were diagnosed with EPTB achieved a 100% favourable outcome, with a classification of ‘treatment completed’ (see Table 2).

**TABLE 2:** TB treatment outcomes of HCWs (No. of HCWs = nine [9])

TYPE OF TB	NO. OF HCWS	TREATMENT OUTCOMES					

		CURE (%)	TREATMENT COMPLETED (%)	TREATMENT FAILURE (%)	TREATMENT INTERRUPTED (%)	DIED (%)	TRANSFER OUT (%)
Smear +ve PTB	7	100	–	–	–	–	–
Smear –ve PTB	0	–	–	–	–	–	–
EPTB	2	–	100	–	–	–	–

## DISCUSSION

Few studies undertaken into the rate of TB among HCWs in different countries have reported treatment outcomes.

In South Africa, Wilkinson and Gilks^11^ studied the impact of the HIV epidemic on the frequency of staff diagnosed with TB in a district hospital in KwaZulu-Natal province between 1991 and 1996. They reported that a total of 22 cases of TB were diagnosed among the staff members, with 18 (82%) of the 22 staff successfully completing their treatment and four (18%) having died. Also in the same province, Naidoo and Jinabhai^12^ studied the incidence of TB, the clinical presentation and the treatment outcomes among HCWs in public sector hospitals in Ethekwini Municipality from January 1999 to June 2004, reported that 583 HCWs were diagnosed with TB during the period, with 40.7% completing their treatment, and 22.2% achieving a cure. Another study undertaken by Eshun-Wilson, Zeier, Barnes and Taljaard^13^ to characterise the occurrence, clinical spectrum and treatment outcomes of TB infection among staff at the Tygerberg Academic Hospital in the Western Cape province, over an 11-year period from 1 January 1996 to 31 December 2006, reported that 130 staff members were diagnosed with TB, with 100 of them being diagnosed with PTB, of which there were 69 sputum smear-positive and 31 sputum smear-negative cases. A cure was achieved in 70.6% of the sputum smear-positive cases. Six of the 130 staff had MDR-TB, of which five were transferred out and one achieved a cure.

In Malawi, one study examined the process of diagnosis and the treatment of smear-positive PTB patients at Queen Elizabeth Central Hospital in Blantyre. The study, which also reported the incidence of TB among nurses working in specific departments of the hospital between 1993 and 1994, reported that 12 nurses were diagnosed and treated for TB during the period, with 11 of the 12 completing their treatment and one having died.^14^ Another study that was undertaken into the incidence of TB among HCWs in the district, government and mission hospitals throughout the country during 1996 reported that 108 HCWs were registered and treated for TB, with 82 (76%) of the 108 completing their treatment and 26 (24%) having died.^15^

In Morocco, a study undertaken by Laraqui, Ottmani, Hammou, Bencheikh and Mahjour^16^into the risk and incidence of TB among HCWs throughout the country between 1994 and 1997 reported that 130 new cases of TB were notified as occurring among HCWs during the period under review. The cohort analysis indicated a mean annual treatment success rate of 89.2%, a failure rate of 0.9%, a loss to follow-up rate of 0.8%, a death rate of 3.8%, and a transfer-out rate of 3.1%.

In British Columbia, Pleszewski and FitzGerald^17^ reported a study in which they compared the clinical features and prevalence of active TB among HCWs with those of the general population in the country between 1991 and 1996. They noted that the treatment completion rate was 84% for both groups.

The abovementioned studies have shown that the treatment outcomes for TB among HCWs vary according to the different localities, with a few places achieving a success rate above 85% and most others achieving a success rate below 85%. The unfavourable outcome of death is not uncommon.

The current study showed no unfavourable treatment outcome among the nine HCWs who were diagnosed with TB during the one-year period. The seven HCWs who were diagnosed with PTB achieved a cure, with the two HCWs who were diagnosed with EPTB successfully completing their treatment, resulting in a treatment success rate of 100% for the nine HCWs concerned.

The 100% treatment success rate achieved may have resulted from consistent adherence to treatment by the HCWs, since none defaulted. Patient adherence to treatment is a key factor in treatment success, especially in the case of standardised short-course first-line anti-TB drug regimens. Such regimens are generally effective in curing TB in new patients, including those with mono- and poly-resistant strains of bacilli.^3, 7 , 8^

The absence of treatment interruption, as well as of any defaulting from treatment by the HCWs concerned, was found to be very encouraging. Such an absence suggests the increased awareness of the HCWs to associated dangers, such as the risk of subsequent development of MDR-TB, which could result in severe morbidity, treatment failure or death.

However, despite the 100% treatment success rate achieved, the current study found that the HCWs did not receive optimal TB care services as only one of the nine HCWs had a TB culture and DST performed at the start of TB treatment. In addition, neither HCW with EPTB had their diagnosis confirmed by means of bacteriological or histopathological method, and the HIV status of seven of the nine HCWs was not known.

TB culture and DST is the recommended method for diagnosing drug-resistant strains of TB.^3, 8, 18^ Routine TB culture and DST at the start of TB treatment is recommended for HCWs, because of their increased risk of contracting drug-resistant TB.^3, 4, 8, 18, 19^ The turnaround time for the test may be up to three months.^8^ The earliest time in which to suspect treatment failure with first-line anti-TB drugs in confirmed new PTB patients is two months, during which period there may be no clinical improvement and/or the sputum smear remains positive for acid-fast bacilli.^3, 7^ Performing TB culture and DST at the start of TB treatment would help to ensure the early diagnosis of drug-resistant TB as the cause of treatment failure. Early arrangements could then be made for the commencement of treatment with the appropriate second-line anti-TB drugs.

The current study found that DST was not performed for the majority of the HCWs. That a treatment success rate of 100% was, nevertheless, achieved only suggests that the HCWs concerned did not have drug-resistant TB, most notably not MDR-TB. Such a finding should, however, not downplay the importance of performing DST at an early stage for those HCWs with TB.

In all patients with EPTB, it is recommended that appropriate specimens be obtained for microscopy from the suspected site of involvement. Where facilities and resources are available, such specimens should be obtained for TB culture and histopathological examination.^4, 19^ The taking of samples is important for HCWs, due to their increased risk of contracting drug-resistant TB, as securing a culture would provide the opportunity for DST to take place.

HIV infection is the most powerful individual risk factor in the case of TB. The HIV epidemic fuels the TB epidemic, and the rate of coexistence of both diseases in individuals is high in areas affected by both the epidemics.^4, 18, 20, 21, 22^ In South Africa, where both epidemics are ongoing, 52% of TB patients who were tested for HIV in 2005 were found to be positive.^5^

HIV-infected HCWs are at increased risk of developing TB if they are exposed to the disease in the workplace.^2^ The drug treatment for TB and the bacteriological response to treatment are the same, irrespective of the HIV status of patients, including that of the HCWs. However, due to the relationship between HIV and TB, voluntary counselling and testing for HIV is recommended as part of the routine management of TB patients. Voluntary counselling and testing offers the opportunity for patients to know their status, as well as for the opportunity for concurrent HIV management in co-infected patients.^4, 7, 18, 19, 23^

The current study has also revealed that the incidence of TB among HCWs at Themba Hospital is close to three times that in the general population of Mbombela South Subdistrict where the hospital is situated. Such a situation calls for urgent investigation and a review of the hospital's TB infection prevention and control plan.

### Limitations

The time period of one year covered by the study was short due to the fact that the occupational health register of Themba Hospital only contained the records of staff members with TB as from December 2006. A follow-up study covering a five year period should give a more reliable picture of TB treatment outcomes among the HCWs at the hospital.

### Conclusion

In conclusion, those HCWs working at Themba Hospital in the Mpumalanga province of South Africa who were diagnosed and treated for TB during 2007 all achieved favourable treatment outcomes. However, in view of the high rate of HIV/ TB co-infection and the increasing problem of the prevalence of drug-resistant TB in the country, the clinical care provided to HCWs with TB working at the hospital should be improved in the areas of routine HIV counselling and testing and the routine early provision of DST. A protocol for the comprehensive management of HCWs with TB is being developed that would be useful not only in the hospital itself, but also in all areas subject to the dual problem of TB and HIV/AIDS, and an increasing prevalence of drug-resistant forms of TB among the general population. The protocol, if used properly by the health care providers concerned, should help to improve the standard of TB care services provided to HCWs with TB.

## References

[1] World Health Organization Guidelines for the prevention of tuberculosis in health care facilities in resource-limited settings Geneva: WHO; 1999.

[2] South African Department of Health The Draft National Infection Prevention and Control Policy for TB, MDR-TB, and XDR-TB Pretoria: Department of Health; 2007.

[3] South African Department of Health DOTS-Plus for standardised management of multidrug-resistant tuberculosis in South Africa: Policy guidelines Pretoria: Department of Health/Medical Research Council; 2004.

[4] South African Department of Health Draft Tuberculosis Strategic Plan for South Africa, 2007–2011 Pretoria: Department of Health; 2006.

[5] World Health Organization Global tuberculosis control: Surveillance, planning, financing Geneva: WHO; 2007.

[6] Weyer K, Brand J, Lancaster J, Levin J, Van der Walt M. Determinants of multidrug-resistant tuberculosis in South Africa: Results from a national survey. SAMJ. 2007;97(11):1120–1128. 18250922

[7] World Health Organization Treatment of tuberculosis: Guidelines for national programmes 3rd ed Geneva: WHO; 2003.

[8] World Health Organization Guidelines for the programmatic management of drug-resistant tuberculosis Geneva: WHO; 2006. 23844450

[9] Gandhi NR, Moll A, Sturm AW, et al. Extensively drug-resistant tuberculosis as a cause of death in patients co-infected with tuberculosis and HIV in a rural area of South Africa. Lancet. 2006;368:1575–1580. 17084757 10.1016/S0140-6736(06)69573-1

[10] South African Department of Health New Tuberculosis Control Programme Drug Regimens Pretoria: Department of Health; 2003.

[11] Wilkinson D, Gilks CF. Increasing frequency of tuberculosis among staff in a South African district hospital: Impact of the HIV epidemic on the supply side of health care. Trans R Soc Trop Med Hyg. 1998;2:500–502. 9861361 10.1016/s0035-9203(98)90889-6

[12] Naidoo S, Jinabhai CC. TB in health care workers in KwaZulu-Natal, South Africa. Int J Tuberc Lung Dis. 2006;10:676–682. 16776456

[13] Eshun-Wilson I, Zeier MD, Barnes J, Taljaard JJ. TB infection among staff at Tygerberg Academic Hospital, South Africa. Sthern Afr J Epid & Inf 2008;23(4):17–19.

[14] Harries AD, Kamenya A, Namarika D et al. Delays in diagnosis and treatment of smear-positive tuberculosis and the incidence of tuberculosis in hospital nurses in Blantyre, Malawi. Trans R Soc Trop Med Hyg. 1997;91:15–17. 9093617 10.1016/s0035-9203(97)90376-x

[15] Harries AD, Nyirenda TE, Banerjee A, Boeree MJ, Salaniponi FM. Tuberculosis in health care workers in Malawi. Trans R Soc Trop Med Hyg. 1999;93:32–35. 10492785 10.1016/s0035-9203(99)90170-0

[16] Laraqui CH, Ottmani S, Hammou MA, Bencheikh N, Mahjour J. Study of tuberculosis in health care workers in the public sector of Morocco. Int J Tuberc Lung Dis. 2001;5:939–945. 11605888

[17] Pleszewski B, FitzGerald JM. Tuberculosis among health care workers in British Columbia. Int J Tuberc Lung Dis. 1998;2:898–903. 9848610

[18] South African Department of Health Mobilising Against Tuberculosis: South African Plan for Tuberculosis Control for 2002 to 2005 Pretoria: Department of Health; 2001.

[19] Tuberculosis Coalition for Technical Assistance International Standards for Tuberculosis Care (ISTC) The Hague: Tuberculosis Coalition for Technical Assistance; 2006.

[20] World Health Organization Strategic framework to decrease the burden of TB/HIV Geneva: WHO; 2002.

[21] World Health Organization TB/HIV: A clinical manual 2nd ed Geneva: WHO; 2004.

[22] World Health Organization Guidelines for implementing collaborative TB and HIV programme activities Geneva: WHO; 2003.

[23] South African Department of Health The Primary Health Care Package for South Africa – a set of norms and standards Pretoria: Department of Health; 2000.

